# Knockdown of yes-associated protein inhibits proliferation and downregulates large tumor suppressor 1 expression in MHCC97H human hepatocellular carcinoma cells

**DOI:** 10.3892/mmr.2015.3257

**Published:** 2015-01-26

**Authors:** CHENG WANG, ZI-MAN ZHU, CHENG-LI LIU, XIAO-JUN HE, HONG-YI ZHANG, JIA-HONG DONG

**Affiliations:** 1 Department of Hepatobiliary Surgery, Air Force General Hospital, PLA, Beijing 100142, P.R. China; 2 Department of Hepatobiliary Surgery, Southwest Hospital, Third Military Medical University, Chongqing 400038, P.R. China; 3 Department of Hepatobiliary Surgery, First Hospital Affiliated to PLA General Hospital, Beijing 100048, P.R. China; 4 Department of Hepatobiliary Surgery, General Hospital of PLA, Beijing 100853, P.R. China

**Keywords:** yes-associated protein, large tumor suppressor 1, hepatocellular carcinoma, cell proliferation, gene therapy

## Abstract

The pathogenesis of hepatocellular carcinoma (HCC) is thought to involve the interaction of numerous genes. Identification of these genes and proteins which regulate liver carcinogenesis is critical for the exploration of novel targeted therapies. Yes-associated protein (YAP) and large tumor suppressor 1 (LATS1) are associated with HCC cells. LATS1 is an upstream inhibitory factor of YAP in the Hippo pathway. The aim of the present study was to measure the expression of LATS1 in Yap-downregulated cancer cells. Immunohistochemistry was used to determine YAP and LATS1 levels in HCC tissue samples. High YAP-expressing cell lines were selected from two human hepatocellular carcinoma cells with different metastatic potential. In addition, changes in cell growth rates and LATS1 expression in human HCC 97H cells, in which YAP had been knocked down using RNA interference (RNAi). The proliferation of cells was evaluated using an MTS assay and changes in the progression of cell division were assessed through cell cycle analysis. Western blot analysis was then used to determine YAP and LATS1 expression levels in 97H cells. The results of the present study demonstrated that overexpression of YAP was negatively correlated with LATS1 expression in HCC cells (P=0.016). Knockdown of YAP using lentivirus-small hairpin (sh)RNA significantly inhibited 97H cell growth; in addition, the downregulation of YAP protein levels (33.4%) was accompanied by downregulation of LATS1 protein levels (68.5%). In conclusion, these results demonstrated that as an inhibitor of YAP, LATS1 was decreased via downregulation of YAP using RNAi. This therefore indicated that the change in YAP levels in HCC cells may regulate LATS1 in a feedback manner.

## Introduction

Hepatocellular carcinoma (HCC) is one of the most prevalent malignant cancers and a major cause of mortality worldwide, most notably in East Asia (1); however, the incidence rate of HCC is rising in Western countries (2). Despite the development of novel therapies for HCC patients, prognoses remain poor; this may be due to the molecular abnormalities of cancer cells. Identification of the genes and proteins which regulate liver carcinogenesis is critical for the exploration of novel targeted therapies for HCC.

Yes-associated protein (YAP), the downstream target molecule of the Hippo signaling pathway, is a transcription co-activator which cooperates with the transcriptional enhancer activator domain factor. Overexpression of YAP has been reported to aberrantly activate the target gene connective-tissue growth factor (3) as well as induce the proliferation of cancer cells (4). As an oncogene (5), YAP protein levels were shown to be elevated and localized in the nuclei of HCC tissues (4). In addition, YAP messenger (m)RNA levels were reported to be significantly elevated in the majority of HCC tissues compared with those in adjacent non-tumor tissues (6,7).

Tumor cell motility is a key determinant of HCC progression and proliferation. YAP has been shown to have a significant role in HCC cell motility via its role in the Hippo signaling pathway. In addition, large tumor suppressor (LATS)1, an inhibitor of YAP activity (8), was found to decrease the motility of human hepatocellular carcinoma HepaRG cells *in vitro* (9). Furthermore, it has been reported that mice deficient in LATS1 developed soft-tissue sarcomas as well as ovarian stromal cell tumors and were highly sensitive to carcinogens (10). These studies therefore suggested that YAP acted as an oncogene, while LATS1 acted as a tumor suppressor gene. It has been hypothesized that mutations associated with LATS1 may occur in numerous HCC cells; therefore, YAP and LATS1 may be promising therapeutic targets for the treatment of HCC.

Two human HCC cell clones with high and low metastatic potential, MHCC-97H (97H) and MHCC-97L (97L), derived from the parental cell line MHCC97, have previously been established (11,12). However, the YAP expression levels and proliferation rates of these two clones of the same genetic background have not been examined.

A previous study based on a Chinese cohort in Hong Kong revealed that YAP was an independent prognostic marker for overall and disease-free survival times in HCC patients (6). Another previous study showed that YAP mRNA and protein levels were significantly higher in HCC tissues compared with those in para-cancerous tissue (PCT) (7). Numerous HCC patients were more amenable to surgery; however, these patients still had a poor prognosis due to early recurrence following partial hepatectomy. YAP levels in the resected HCC tissue may therefore provide a valuable indicator for effective follow-up management. Further studies on regulation of the Hippo pathway may enhance understanding of hepatocarcinogenesis; in addition, the development and use of a targeted therapy against the YAP gene may enable long-term survival for patients with HCC.

Among the above-mentioned issues, it is important to confirm whether knockdown of YAP using RNAi significantly inhibited liver cancer cell growth; therefore, as YAP was found to be associated with LATS1 in the Hippo pathway, the aim of the present study was to measure the expression of LATS1 in cancer cells in which YAP was downregulated.

## Materials and methods

### Patients and specimens of HCC

A total of 40 cases of HCC, and 10 cases of PCT were used in the present study. All tissues were obtained from the patients which received curative hepatectomy surgery at the Air Force General Hospital (Beijing, China) between January 2010 and June 2013. All patients were diagnosed with pathological primary HCC and none of the patients had received any radiotherapy or chemotherapy prior to surgery. The PCT was dissected at a margin >1 cm from the tumor edge. Normal liver samples were taken from benign lesions.

All clinical procedures in the present study were approved by an institutional review board of the Air Force General Hospital (Beijing, China) prior to patient enrollment. Written informed consent was obtained from each patient prior to the collection of these tissues.

### Generation of HCC tissue array

A tissue microarray (TMA) was constructed from formalin-fixed (10% paraformic aldehyde; Sigma-Aldrich, St. Louis, MO, USA), paraffin- embedded (Weiqiboxing Co., Wuhan, China) tissue blocks. Two core needle samples, 1.2 mm from each specimen, were obtained from morphologically representative tumor areas of each donor tissue paraffin block. Xylene (Beijing Chemwork, Beijing, China) was used for dewaxing, graded ethanol (100% 10 sec and 95% 10 sec; Hongziweida Co., Beijing, China) was used for rehydration of the samples and neutral balsam (Sinopharm Chemical Reagent Co., Ltd, Shanghai, China). Hematoxylin and eosin staining (Berlin Biological, Beijing, China) of the TMAs was performed in order to verify the histopathological findings.

### Cell lines

HCC cell lines 97H (higher metastatic potential) and 97L (lower metastatic potential) were purchased from the Cell Bank of Shanghai Institute (Shanghai, China; these cell lines were established in nude mice). All cells were cultured in Dulbecco’s modified Eagle medium with 10% fetal bovine serum supplemented with 100 U/ml penicillin and 100 *μ*g/ml streptomycin.

### Immunohistochemistry (IHC) and immunocytochemistry (ICC)

Expression levels of YAP and LATS1 were measured using IHC in a retrospective cohort of liver cancer and matched adjacent non-tumor tissue samples from HCC patients following liver resection. The expression of YAP was also measured using ICC in MHCC97H cells.

Paraffin-embedded tissue blocks were cut into 4-*μ*m sections and each was baked at 60°C for 2 h prior to deparaffinization. Antigen retrieval was achieved through microwave exposure (Milk 700 W; Galanz, Guangdong, China) at 100°C in citrate buffer (0.01 M; Bioss, Inc., Wuhan, China) for 15 min. Following immersion in 3% hydrogen peroxide (Lircon, Sandong, China) for 20 min, the sections and cells were incubated with goat serum (Yuanye Co., Shanghai, China) for 1 h and then incubated with YAP (H-125) rabbit polyclonal immunoglobulin (Ig)G (1:80; sc-15407; Santa Cruz Biotechnology, Inc., Dallas, TX, USA), LATS1 rabbit polyclonal antibodies (1:80; 17049-1-AP; Proteintech Group, Chicago, IL, USA), anti-phospho-LATS1 (Ser909) rabbit polyclonal antibodies (1:80; bs-3246R; Bioss, Inc., Wuhan, China) and GAPDH antibody (1:3,000; GenScript, Piscataway, NJ, USA) at 4°C overnight. Following four washes in phosphate-buffered saline with Tween 20 (Sigma-Aldrich), sections were incubated with peroxidase-conjugated goat anti-rabbit/mouse IgG secondary antibody (K5007 Bottle A, Dako REAL™ EnVision™; Dako, Glostrup, Denmark) for 1 h at 37°C. Visualization was performed using a diaminobenzidine chromogen kit (K5007 Bottles B and C, Dako REAL™ Substrate Buffer and Dako REAL™ DAB+ Chromogen; Dako). Sections were then counterstained with hematoxylin, dehydrated and mounted.

Experiments were repeated twice and the percentage of tumor cells or hepatocytes with obvious staining in the cytoplasm or nucleus were determined in all optical fields of the slices (BX51TR; Olympus Corp., Tokyo, Japan). A percentage >10% was considered to indicate positive immunoreactivity. Two independent, blinded investigators examined all tumor slides at random.

### Lentivirus production and transduction

The sequence for targeting the YAP gene was selected using the short hairpin RNA (shRNA) Clone Library [http://cgap.nci.nih.gov/RNAi/RNAi2]. The effective target point sequence of YAP gene YAP1 NM_001130145 Human, 5′-CATTGCTGCTGTTAATGTA-3′, was selected. A lentivirus (pMagic 4.1) construct was provided by Shanghai Sunbio Medical Biotechnology Co. Ltd (SB1262-C; Shanghai, China).

97H cells were transduced with lentiviral vectors and control lentivirus in complete medium containing polybrene (8 mg/ml; Sigma-Aldrich). At 72 h following the first transfection, total RNA and protein were extracted from cells with radioimmunoprecipitation lysis buffer (Beyotime Institute of Biotechnology, Haimen, China) with a protease inhibitor (Roche, Basel, Switzerland) and reverse transcription quantitative polymerase chain reactions (RT-qPCR) as well as western blot analysis were performed in order to evaluate the inhibitory effects of YAP.

### Flow cytometric analysis of the cell cycle

Cells were harvested during the exponential growth phase and single-cell suspensions containing 1×10^6^ cells were fixed with 75% ethanol (Hongziweida Co.) for 48 h at 4°C. The cell cycle was monitored using propidium iodide (PI; 50 *μ*g/ml; Sigma-Aldrich, St. Louis, MO, USA) staining. The fluorescence of DNA-bound PI in cells was measured using a flow cytometer (FACS101; BD Biosciences; Franklin Lakes, NJ, USA).

### In vitro cell growth assay

Cell Titer 96 AQueous One Solution Cell Proliferation Assay (3582; Promega Corp., Madison, WI, USA) containing 3-(4,5-dimethylthiazol-2-yl)- 5-(3-carboxymethoxyphenyl)-2-(4-sulfophenyl)-2H-tetrazolium (MTS) was used to assess cell proliferation, as previously described (13).

A cell suspension was prepared at a concentration of 1×10^4^/ml. Aliquots (100 *μ*l) were dispensed into 96-well microtiter plates and incubated for five days. MTS assays were performed by adding 20-*μ*l MTS reagent into each well and incubating the plate at 37°C for 3 h in a humidified, 5% CO_2_ atmosphere. The optical density (OD) of each well was measured daily using a microplate reader (550; Bio-Rad Laboratories, Inc., Hercules, CA, USA) at 490 nm; all OD values were calculated relative to OD at day 1.

### RT-qPCR

Total RNA was extracted using TRIzol (1382739; Invitrogen Life Technologies, Carlsbad, CA, USA). Total RNA concentrations were measured using spectrophotometry (DU-7; Beckman Coulter, Inc., Fullerton, CA, USA) and then 300 ng total RNA from each sample was reverse-transcribed to complementary (c)DNA using the PrimeScript RT reagent kit (DRR037A; TaKaRa Bio, Inc., Shiga, Japan). cDNA was amplified by qPCR using SYBR Green (DRR041A; TaKaRa Bio, Inc.). The amplification process was applied in a LightCycler 2.0 system with LightCycler software version 3.5 (Roche). Primer details are listed in Table I. Amplification conditions were as follows: 95°C for 30 sec, then 40 cycles at 95°C for 5 sec and 64°C for 30 sec. A dissociation procedure was performed to generate a melting curve for confirmation of amplification specificity. The analysis of PCR products by 1% agarose gel electrophoresis (Aladdin, Shanghai, China) confirmed amplification specificity. Primers were designed by the Sangon Biotech Co. (Shanghai, China). Primer specificity was confirmed using Primer BLAST software (National Center for Biotechnology Information, Bethesda, MD, USA). Relative quantification was accomplished using a double standard curve method or the 2^−ΔΔCt^ method, as previously described (14). Ct values of the samples were normalized to the appropriate endogenous housekeeping gene GAPDH. Each measurement was repeated in triplicate.

### Western blot analysis

Cells were lysed using radioimmunoprecipitation buffer (P0013B; Beyotime Institute of Biotechnology) with a protease inhibitor (11873580001; Roche). A bicinchoninic acid assay (P0010; Beyotime Institute of Biotechnology) was used to determine protein concentrations, as previously described (15). A total of 40 *μ*g protein was boiled for 10 min prior to loading and then separated using 10% SDS-PAGE. Proteins were then transferred onto nitrocellulose membranes (0.45 *μ*m; Whatman Ltd, Clifton, NJ, USA) using a semi-dry transfer system (Bio-Rad Laboratories, Inc.). Following blocking with 5% bovine serum albumin (HyClone, GE Healthcare Life Sciences, Logan, UT, USA), the membrane was probed with YAP (H-125) rabbit polyclonal immunoglobulin (Ig) G (1:300), LATS1 rabbit polyclonal antibody (1:200), β-actin antibody (1:500, Immunocreate, Hoover, AL, USA) and GAPDH antibody (1:3,000; GenScript). Horseradish peroxidase (HRP)-conjugated goat anti-rabbit polyclonal secondary antibody (1:40,000; 31460, Thermo Fisher Scientific, Waltham, MA, USA) and HRP-conjugated goat anti-mouse polyclonal IgG (1:40,000; 115-035-003, Jackson Immunoresearch Laboratories Inc., West Grove, PA, USA) were then used. Protein band signals were amplified using enhanced chemiluminescence detection reagents (34079; Thermo Fisher Scientific). Protein levels were determined semi-quantitatively using Quantity One 4.4.0 imaging software (Bio-Rad Laboratories, Inc.).

### Statistical analysis

The Pearson Chi-Square test and the independent sample *t*-test were used to analyze differences between values. All statistical analyses were performed using the SPSS 13.0 software (SPSS, Inc., Chicago, IL, USA). P<0.05 was considered to indicate a statistically significant difference between values.

## Results

### Compared with adjacent tissues, HCC tissues express higher levels of YAP and lower levels of LATS1

**Figure 1:**
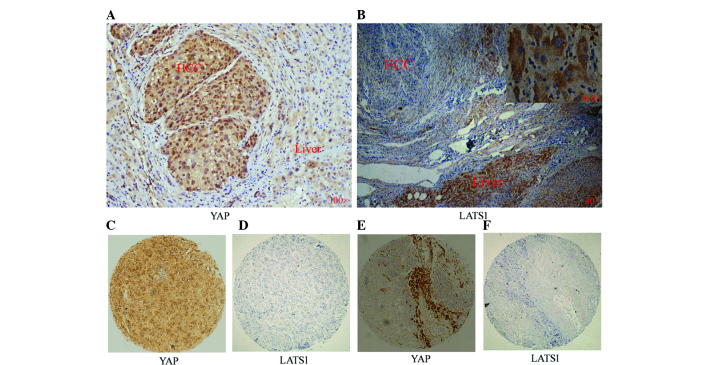
Expression of YAP and LATS1 in HCC and PCT tissues. Representative images of (A) YAP staining (magnification, ×100) or (B) LATS1 staining in HCC (magnification, ×40) and PCT (magnification, ×40 and ×400) as detected by immunohistochemistry. (C and D) Identical HCC tissue slice stained for YAP and LATSI, respectively. (E and F) Another identical HCC tissue slice stained for YAP and LATSI, respectively (magnification, ×40). YAP, yes-associated protein; LATS, large tumor suppressor 1; HCC, hepatocellular carcinoma; PCT, para-cancerous tissue.

YAP and LATS1 protein expression levels were investigated in 40 HCC tissue samples and 10 PCT tissue samples using IHC. As shown in Fig. 1A, the positivity rate of YAP protein in HCC tissue was significantly higher compared with that of PCT (72.5 and 20.0%, respectively; P=0.002). In addition, compared with adjacent tissues, HCC tissues expressed higher levels of YAP protein; furthermore, YAP was found to be concentrated in the nuclei of HCC cells. The positivity rate of LATS1 protein in HCC tissue was significantly lower compared with that of PCT (17.5 and 60.0%, respectively; P=0.019). Compared with the adjacent tissues, HCC tissues had lower levels of LATS1 protein, which was predominantly located in the cytoplasm of PCT cells (Fig. 1B).

### In HCC tissues, high levels of YAP are usually accompanied by low levels of LATS1

The results of the present study also showed that overexpression of YAP occurred primarily in HCC tissues in which LATS1 expression was low (Fig. 1C–F). The positive rate of YAP expression demonstrated a negative correlation with that of LATS1 expression in HCC (72.5 and 17.5%, respectively; P=0.016) (Table II).

### MHCC97 cells with high YAP expression have an increased proliferative capacity

**Figure 2:**
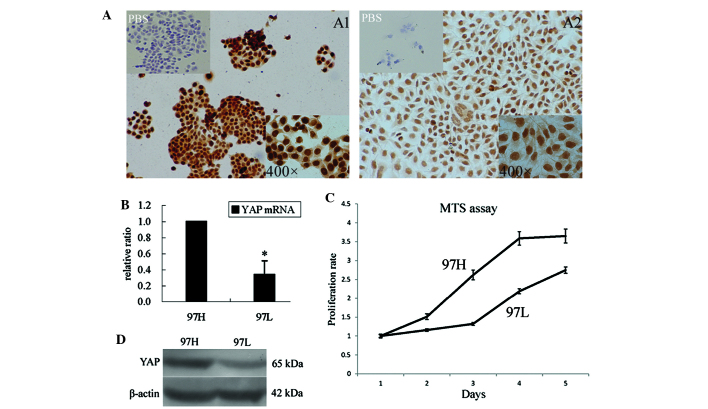
Expression of YAP in 97H and 97L cell lines. (A) Immunocytochemical staining of YAP in 97H and 97L cells, respectively (magnification, ×100). Negative controls were treated with PBS instead of the first antibody. (B) YAP mRNA levels in 97H and 97L cell lines as determined by reverse transcription quantitative polymerase chain reaction (^*^P=0.021; n=3). Relative quantification was accomplished using a double standard curve method and normalized to GAPDH mRNA. (C) Cell proliferation rates were measured using an MTS assay. Values are presented as the mean ± standard deviation. Proliferation rates are expressed relative to that of day one. (D) YAP protein levels in 97H and 97L cells as determined by western blot analysis. β-actin levels were used to normalize the data. YAP, yes-associated protein; 97H/L, MHCC97H/L; PBS, group treated with phosphate-buffered saline instead of first antibody.

YAP expression in two liver cancer cell lines with different metastatic properties were examined using ICC and western blot analysis (Fig. 2A and D, respectively). The results showed that 97H cells aggregated during growth, whereas 97L cells spread out during growth. 97H cells, which are highly metastatic, expressed higher protein levels of YAP compared with those of 97L cells. As shown in Fig. 2B, 97H cells expressed higher levels of YAP mRNA compared with those in 97L cells (P=0.021). Of note, the results of the MTS assay showed that the proliferation of 97H cells was significantly higher compared with that of 97L cells (Fig. 2C); however, 97 L cells also grew rapidly. The proliferation multiples of 97H and 97L on day five were 3.65±0.06 and 2.75±0.03, respectively (P<0.05). Therefore, the proliferation activity of these liver cancer cells paralleled the expression levels of YAP.

### RNAi-mediated YAP gene silencing inhibits 97H cell proliferation and cell cycle

**Figure 3:**
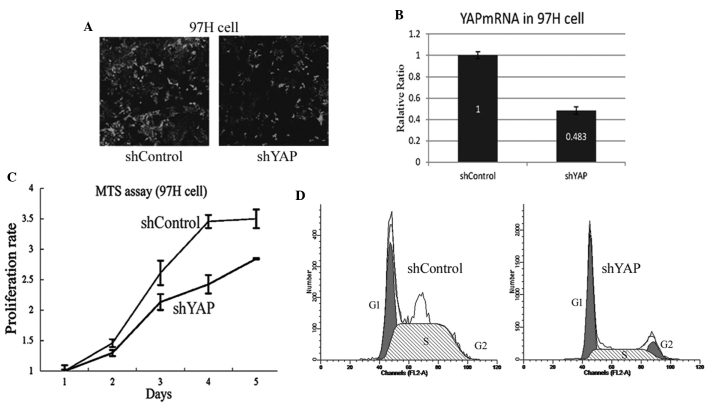
Effects of lentivirus-mediated RNA interference on YAP expression and cell proliferation. (A) Fluorescence photomicrographs of 97H cells demonstrate the transduction efficiency of shControl and shYAP. Images were captured at 72 h following transfection with identical cell densities in six-well plates. Cell counts in shYAP wells were lower compared with those of the shControl wells. (B) Effects of knockdown of YAP mRNA on 97H cells, as determined by reverse transcription polymerase chain reaction. Relative quantification of mRNA was accomplished by the 2^−ΔΔCt^ method. (C) Cell proliferation rate was measured using an MTS assay. Results are presented as the mean ± standard deviation. Proliferation rates are expressed relative to that of day one. (D) Effects of YAP knockdown on the cell cycle progression as detected by flow cytometric analysis. YAP, yes-associated protein; 97H, MHCC97H; shControl, control short hairpin RNA-transfected cells; shYAP, YAP short hairpin RNA-transfected cells.

**Figure 4:**
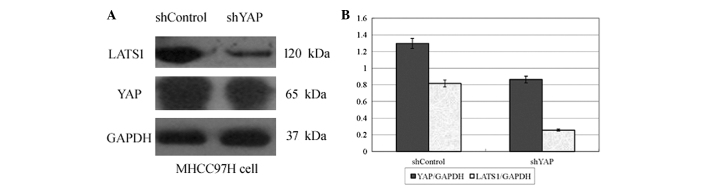
Effects of lentivirus-mediated RNA interference on YAP and LATS1 expression in 97H cells. (A) Effects of knockdown of YAP expression on YAP and LATS1 protein as determined by western blot analysis in shControl- and shYAP-transfected cells. (B) Quantitative analysis of YAP and LATS1 protein bands relative to GAPDH using Quantity One imaging software. YAP, yes-associated protein; LATS1, large tumor suppressor 1; 97H, MHCC97H; shControl, control short hairpin RNA-transfected cells; shYAP, YAP short hairpin RNA-transfected cells.

Lentivirus-mediated RNAi was used to efficiently downregulate YAP expression in 97H cells as shown in the fluorescence photomicrograph images (Fig. 3A), as well as by the significantly decreased mRNA (Fig. 3B) and protein (Fig. 4A) levels of YAP following shRNA YAP (shYAP) transfection compared with that of the shRNA control-transfected (shControl) cells. The results showed that cell proliferation was significantly suppressed and cell cycle progression was altered in shYAP-transfected 97H cells (Fig. 3C and D). The proliferation multiples of shYAP-transfected cells and shControl cells on day four following transfection were 2.42±0.15 and 3.45±0.11, respectively. Cell cycle analysis of 97H cells using flow cytometry revealed that the proportion of shYAP-transfected cells in the G1, G2 and S phases were 50.33, 13.94 and 35.73%, respectively, whereas the proportion in each cell cycle phase in shControl cells were 33.67, 0.94 and 65.39%, respectively; the differences between the two groups were significant for all phases (P<0.001). The proportion of cells in the G1 and G2 phase was significantly increased in shYAP-transfected cells, whereas the proportion cells in S phase was decreased as compared with populations of shControl cells. This therefore indicated that YAP silencing induced G1 and G2 phase arrest as well as a decrease of cells in S phase.

### YAP and LATS1 are downregulated in shYAP-transfected 97H cells

As shown in Fig. 4, western blot analysis revealed that protein levels of YAP and LATS1, the main upstream regulator of YAP, were significantly decreased in shYAP-transfected cells compared with those in the shControl group. Downregulation of YAP protein levels was accompanied by downregulation of LATS1 protein levels (33.4 and 68.5%, respectively; P<0.05). These results indicated that changes in YAP gene expression affected the expression of LATS1 through a positive regulatory association between YAP and LATS1. Following YAP mRNA downregulation, YAP protein levels decreased and in turn, the proliferation of cancer cells decreased; therefore, by reducing the inhibition of YAP, cancer cells decreased the expression of LATS1, demonstrating a novel negative feedback loop in the shYAP cells.

## Discussion

YAP is a transcriptional co-activator which has been shown to be a regulator of cell growth in the Hippo signaling pathway (1). It has been previously confirmed that YAP was phosphorylated by LATS1, the upstream factor in the Hippo pathway (8,16), which was reported to be able to bind to and phosphorylate transcription regulator YAP in cell culture and *in vivo* (8). Phosphorylation of YAP was found to be associated with its export from the nucleus to the cytoplasm, where it downregulates growth-associated genes (4,8).

YAP mRNA and protein levels were previously reported to be higher in major HCC than in para-cancerous tissue (7). One study showed that following YAP1 transformation, a non-tumorigenic hepatocyte cell line formed visible tumors in a nude mouse model; however, this cell line was unable to form subcutaneous tumors prior to transformation (17). These results indicated that YAP may be involved in the pathogenesis of HCC. In addition, numerous studies have provided evidence linking YAP gene expression to tumorigenicity in several solid cancers (4,18,19). Nuclear overexpression of YAP was reported to contribute to the growth of pulmonary adenocarcinoma (20) and the widespread upregulation of YAP in a variety of tumor types further suggested that the YAP gene may represent a gene which allows cancer cells to evade the effects of growth inhibition. Furthermore, excessive proliferation may result in the evasion of intrinsic size-control mechanisms, which may lead to gradual increases in tumor volume. These findings indicated that YAP may contribute to multiple aspects of tumor progression and neoplasia; therefore, YAP may be a potential therapeutic target molecule for HCC intervention. By contrast, LATS1 was found to negatively regulate the transcriptional and transformational functions of YAP through inhibiting its nuclear translocation (8); therefore, LATS1 may also be a potential therapeutic target molecule for the treatment of HCC.

In order to study the association between YAP and LATS1 in mammals, the present study aimed to investigate YAP and LATS1 protein levels in 40 cases of HCC tissue, and 10 cases of PCT using IHC. The results of the present study were consistent with those of previous studies (6,9), which showed that YAP protein was overexpressed in HCC samples compared that of PCT; in addition, the present study revealed that the majority of normal liver tissues exhibited extensive activation of LATS1 protein, which was not observed in most HCCs. Exactly the opposite was found for the expression of YAP. Another previous study showed that western blot analysis was able to detect LATS1 in all normal liver tissues, whereas it was only detected in three out of seven HCC samples (9), which indicated that LATS1 loss may be involved in the development of liver cancer; therefore, a YAP-targeted therapy for liver cancer must focus on LATS1.

In the present study, YAP-positive HCC cells were found to have low LATS1 expression. It has already been confirmed that YAP was phosphorylated by LATS1, the upstream factor in the Hippo signaling pathway (8,16). This therefore indicated that LATS1 inactivation decreased YAP phosphorylation, leading to the overexpression of YAP in HCCs.

A previous study using MTT assays showed that following YAP1 transformation, non-tumorigenic hepatocyte cell clones demonstrated faster growth rates compared with those of cells transfected with empty vector controls (17). This therefore indicated that YAP may be a determinant of liver cancer cell proliferation. In the present study, cell growth curves revealed that 97H cells with higher levels of YAP gene expression exerted a significantly increased proliferative activity compared with that of 97L cells with lower levels of YAP, indicating that high expression of YAP in liver cancer cells may induce a high proliferative activity. Therefore, in the present study, 97H cells were selected for RNA interference experiments.

The results of the present study demonstrated that shRNA-mediated downregulation of YAP expression resulted in a decrease in the number of cells in S-phase as well as significant cell growth inhibition *in vitro*. This further indicated that HCC cell growth was associated with YAP expression levels. Further analysis revealed that downregulation of YAP in 97H cells was accompanied by a decrease in the expression of LATS1. This therefore led to the hypothesis that a negative feedback control mechanism existed in the Hippo pathway of 97H cells, which may compensate for RNA interference against YAP in 97H hepatocellular carcinoma cells.

LATS1 has a critical role in the Hippo pathway; however, the mechanisms by which LATS1 is regulated at the protein level remains to be fully elucidated (21). Certain positive regulators of LATS1, including macrophage-stimulating protein 1/2 (8), hMOB1 (22) and kidney and brain expressed protein (23) have been identified in previous studies, as well as negative regulators of LATS1, including E3 ubiquitin ligase Itch (24) and WW domain-containing E3 ubiquitin protein ligase 1 (25). These proteins have been suggested to be involved in the negative feedback control mechanism between YAP and LATS1; however, further studies are required in order to clarify this hypothesis.

In the present study, a gradient of YAP expression in PCT and HCC suggested that the occurrence, development and progression of HCC was associated with increased YAP expression. A previous study demonstrated that overexpression of YAP was accompanied by high levels of YAP mRNA in HCC tissues (7). If the high levels of YAP were due to overexpression of the YAP gene, and not LATS1 inactivation, the normal function of LATS1 may be to feedback and suppress the production of YAP protein. However, in the present study, LATS1 protein levels were not found to be increased; by contrast, LATS1 expression was significantly decreased in HCC cells and YAP-positive HCCs expressed low levels of LATS1.

In conclusion, the results of the present study demonstrated that there was a negative correlation between YAP and LAST1 expression in HCC tissues. In addition, RNAi-mediated YAP gene silencing inhibited 97H cell proliferation and cell cycle progression. Furthermore, LATS1 protein levels were markedly decreased in 97H cells in which YAP was downregulated. These results provided additional evidence that LATS1 levels may compensate for the effects of the RNA interference against YAP in MHCC97H cells. Therefore, these findings may form the basis for YAP inhibition and LATS1 stimulation as targeted therapies for the future treatment of HCC.

## Figures and Tables

**Figure 5:**
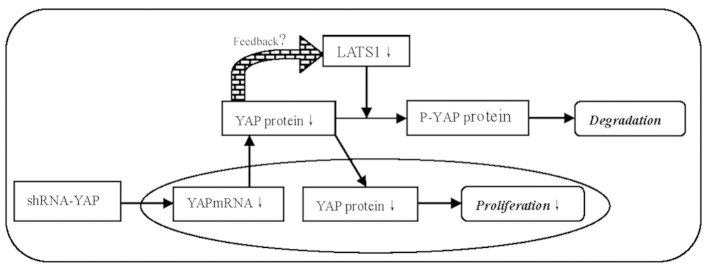
Schematic showing the partial Hippo pathway in HCC cells as determined by the results of the present study. The corresponding proteins and genes in HCC cells are indicated by rectangles. ‘Degradation’ and ‘proliferation’ are indicated by rounded rectangles. A thick arrow represents a possible feedback mechanism between YAP and LATS1. HCC, hepatocellular carcinoma; YAP, yes-associated protein; LATS1, large tumor suppressor 1; shRNA, short hairpin RNA; P-, phosphorylated.

**Table I:** Primers used for reverse transcription quantitative polymerase chain reaction.

Gene	Primer	Length (bp)
Yes-associated protein (Human)	Forward, 5′-ACCCACAGCTCAGCATCTTCG-3′ Reverse, 5′-TGGCTTGTTCCCATCCATCAG-3′	257
GAPDH (Human)	Forward, 5′-AGAAGGCTGGGGCTCATTTG-3′ Reverse, 5′-AGGGGCCATCCACAGTCTTC-3′	258

**Table II:** Association between YAP and LATS1 in hepatocellular carcinoma.

		YAP		χ^2^	P-value
		P	N		
LATS1	P	2	5	5.759	0.016
	N	27	6		

Chi-square (χ^2^) tests with continuity correction were used to demonstrate a negative correlation between YAP and LATS1. All statistical tests were two-sided. YAP, yes-associated protein; LATS, large tumor suppressor 1; P, positive; N, negative.
